# An *in silico* survey of *Clostridioides difficile* extrachromosomal elements   

**DOI:** 10.1099/mgen.0.000296

**Published:** 2019-09-17

**Authors:** Bastian V. H. Hornung, Ed J. Kuijper, Wiep Klaas Smits

**Affiliations:** ^1^​ Department of Medical Microbiology, Leiden University Medical Center, PO Box 9600, 2300RC, Leiden, The Netherlands; ^2^​ Netherlands Centre for One Health, The Netherlands; ^3^​ Centre for Microbial Cell Biology, Leiden, The Netherlands

**Keywords:** *Clostridium difficile*, *Clostridioides difficile*, plasmids, sequence analysis

## Abstract

The Gram-positive enteropathogen *Clostridioides difficile* (*Clostridium difficile*) is the major cause of healthcare-associated diarrhoea and is also an important cause of community-acquired infectious diarrhoea. Considering the burden of the disease, many studies have employed whole-genome sequencing of bacterial isolates to identify factors that contribute to virulence and pathogenesis. Though extrachromosomal elements (ECEs) such as plasmids are important for these processes in other bacteria, the few characterized plasmids of *C. difficile* have no relevant functions assigned and no systematic identification of plasmids has been carried out to date. Here, we perform an *in silico* analysis of publicly available sequence data to show that ~13 % of all *C. difficile* strains contain ECEs, with 1–6 elements per strain. Our approach identifies known plasmids (e.g. pCD6, pCD630 and cloning plasmids) and six novel putative plasmid families. Our study shows that plasmids are abundant and may encode functions that are relevant for *C. difficile* physiology. The newly identified plasmids may also form the basis for the construction of novel cloning plasmids for *C. difficile* that are compatible with existing tools.

## Data Summary

Sequence information from this study has been deposited in the European Nucleotide Archive under BioProject accession number PRJEB32360 (accession numbers LR594541–LR594546 and LR595854).

**Impact Statement**

Genomic studies of the main cause of nosocomial infectious diarrhoea, *Clostridioides difficile (Clostridium difficile)*, to date have focussed on the analysis of the chromosome and core genome. Despite the fact that plasmids in this organism were first demonstrated decades ago, little is known about their abundance and role in *C. difficile* physiology. Therefore, we analysed publicly available genome sequences for putative extrachromosomal elements, which includes plasmids. We have found that plasmids may be common and encode functions that may be relevant for pathogenesis. Our work could also form the basis for the development of novel plasmid vectors for the genetic manipulation of *C. difficile *.

## Introduction

*Clostridioides difficile* (* Clostridium difficile *) [1] is a Gram-positive, endospore forming, anaerobic bacterium. It is an opportunistic pathogen in humans, and is the causative agent of most cases of antibiotic-associated diarrhoea [2]. In addition, in recent years, the bacterium has been increasingly found in cases of infectious diarrhoea that cannot be linked to healthcare exposure [2]. *C. difficile* infections can be refractory to antimicrobial therapy and even when an initial cure is observed, relapses are frequent [2]. Typing methods for *C. difficile* include (capillary) PCR ribotyping, multilocus sequence typing (MLST) and SNP typing after whole-genome sequencing [3]. Since the beginning of the 21st century, an increase in *C. difficile* infections due to epidemic types such as PCR ribotype 027 and 078 has been noted [4, 5]. Although the molecular mechanisms underlying the epidemicity are poorly understood and remain under debate [6], robust toxin production and sporulation [7], altered surface properties [8], resistance to antimicrobials [5, 9] and an increased ability to metabolize certain sugars [10] have been implicated.

In other organisms, the contribution of plasmid-encoded functions to virulence and pathogenesis is well-documented [11–13]. By contrast, only a limited number of plasmids have been identified in *C. difficile* and all of these are cryptic, i.e. no traits have been associated with plasmid carriage. Commonly used cloning vectors for *C. difficile* make use of a replicon derived from the 6.8 kb plasmid pCD6 [14]. The reference strain 630 contains a single plasmid, pCD630, that is part of a larger family of 7.8–11.8 kb plasmids [15, 16]. In addition, recently several large (>42 kb) plasmids were described [17]. Nevertheless, earlier work supports the notion that plasmids may be common in *C. difficile* [18, 19]. It is also possible, however, that other extrachromosomal elements (ECEs) were detected in these studies: both conjugative transposons and phages can exist as circular dsDNA intermediates [20–23].

Several approaches to identify plasmids from next-generation sequencing data have been described, based on sequence homology, coverage, contig interactions or machine learning approaches [24]. None of these have been applied for a systematic investigation of ECEs in *C. difficile *.

Here, we used a bioinformatic approach to identify ECEs in publicly available *C. difficile* whole-genome sequence data. Based on our analysis, we expect that most of these elements represent plasmids, suggesting an as yet untapped potential for virulence determinants, or a source for genetic engineering of diverse *C. difficile *.

## Methods

### Detection of putative ECEs in public databases

**Fig. 1.**
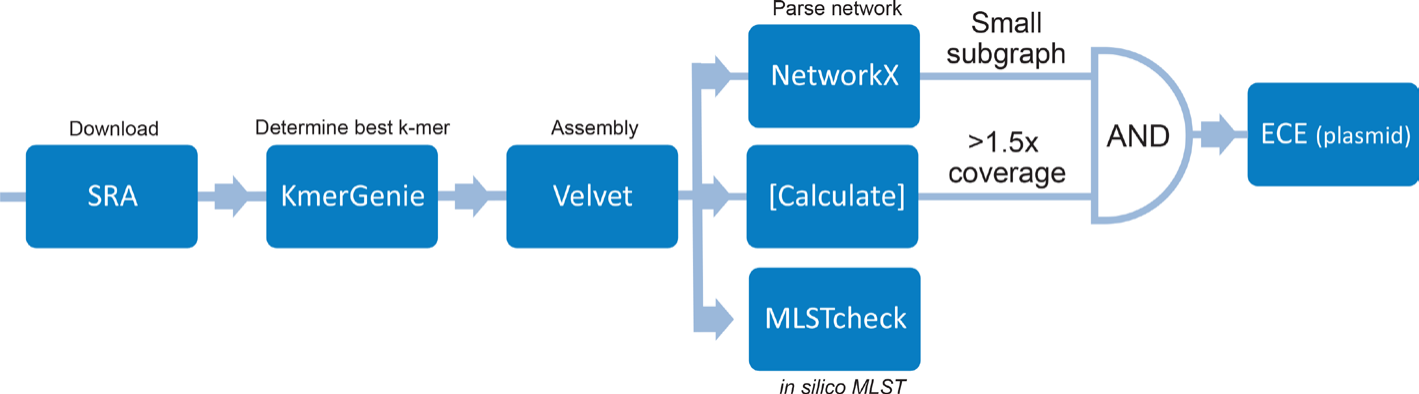
Schematic representation of the approach for the *in silico* identification of ECEs in *C. difficile *.

To identify ECEs in a high-throughput manner, we implemented an approach comparable to PLACNETw [25, 26], a graph-based tool for the reconstruction of plasmids from next-generation sequence paired-end datasets (Fig. 1). This method employs the connections between all contigs of a given sequence dataset to identify contigs/contig groups that are not connected to the main group of contigs that is assumed to represent the chromosome. Additionally, the read coverage of these contigs is compared to that of the chromosome and only elements with a coverage exceeding that of the chromosome are considered ECEs. This could in theory identify plasmids, phages or conjugative transposons that are present in multiple copies per chromosome equivalent and are not integrated into the chromosome at the time of DNA isolation. In short, 5403 public paired-end Illumina datasets were downloaded from the National Center for Biotechnology Information (NCBI) [for accession numbers see Table S1 (available with the online version of this article); accessed on 07/09/2017]. All samples were downloaded with Eutils prefetch [27] and converted with fastq-dump from the SRA toolkit v2.8.2–1. The optimal kmer was predicted by kmergenie v1.6741 [28] on the interleaved fastq files and the assembly was performed with Velvet v1.2.10 [29]. Afterwards, the assembly graph from the Velvet output was parsed into a graph with the Python NetworkX library v1.11 [30]. To calculate size and coverage, all headers in the Velvet assembly were parsed into the network. The biggest component (based on size in bases) was considered to be the genome, and mean coverage was estimated by averaging the coverage of all contigs over the amount of contigs. All other network components were considered to belong to the chromosome if their coverage did not exceed 1.5 times the coverage of the chromosome.

To reduce the number of false-positive identifications, in a second step the coverage was adjusted for the number of base pairs instead of the number of contigs. Furthermore, a blast search (v2.7.1) was performed against the chromosome of *C. difficile* 630 [15], and all components with more than 50 % genomic content were regarded as belonging to the chromosome. Additionally, a blast search was performed with all identified sequences against the NCBI plasmid database ([27]; downloaded 11/09/2017).

To detect homology between the assembled sequences, the average nucleotide identity (ANI) was calculated between all plasmids with pyANI v0.2.7 [31]. The following known plasmids were included as well: pCD6 (AY350745.1), pCD630 (AM180356.1), pCD-WTSI1 (MG019959.1), the plasmid from *C. difficile* strain BI1 (FN668942.1), the big plasmids 1 and 2 of strain FDAARGOS_267 (NZ_CP020425.1 and NZ_CP020426.1), pAK1 and pAK2 (NZ_CP027015.1 and NZ_CP027016.1), pHSJD-312 (MG973074.1), pCd13_cfrC (MH229772.1), LIBA6289 (MF547664.1), pZJCDC-S82 (JYNK01000020.1), *C. difficile* strain CD161 plasmid unnamed1 and 2 (CP029155.1 and CP029156.1), *C. difficile* strain CDT4 plasmid unnamed1 (CP029153) and 25 described phages [20]. To determine the exact grouping, a further clustering analysis was performed. For each group, one representative was chosen based on the results of the pyANI analysis [phiX174, phiCD38-2, phiCD119, pCD-WTSI1, ERR1015479 plasmid 3 (ECE1), ERR251819 plasmid 2 (ECE2), ERR125924 plasmid 2 (ECE3), ERR347487 plasmid 2 (ECE4), ERR340291 plasmid 2 (ECE5) and ERR251831 plasmid 1 (ECE6), as well as ERR125492 plasmid 1 (pCD-SMR), ERR247053 plasmid 1 (aminoglycoside resistance) and ERR125936 plasmid 1 (bacteriocin plasmid)]. Based on the ANI values, a hierarchical clustering with complete linkage (Conda v4.5.11 [32, 33], Python v2.7 [34], SciPy v1.0.0 [35]) was performed with these references and each plasmid. Each plasmid was assigned a type corresponding to its closest neighbour, unless the closest neighbour could not be exactly determined, or the branch rooted deeper in the tree.

Detailed comparative analysis of these sequences was performed with Mauve v2.3.1 [36] and blast [37]. All blast searches within this project were performed with the parameters –evalue 0.0001 and –culling_limit 1, unless otherwise mentioned. Sequence typing of the assembled genomes was performed with MLSTcheck v2.1 [38].

Contamination checking of the assembled genomes was performed with Mash v2.0 [39] (with default parameters). Genomes were considered contaminated if any match with an *E* value bigger than zero was present, which did not contain the words ‘* Clostridioides difficile *’, ‘* Clostridium difficile *’, ‘* Peptoclostridium difficile *’, ‘*Clostridium phage*’, ‘Enterobacteria phage phiX174’ or ‘* Clostridium* sp. HMSC’. Entries containing the word ‘plasmid’ were ignored.

### Plasmid annotation

Annotation was performed with another in-house pipeline. This pipeline used Prodigal version v2.6.3 (with –meta option) [40] for gene calling, RNAmmer v1.2 [41] for rRNA prediction, Aragorn 1.2.38 [42] for tRNA prediction and the CRISPR recognition tool v1.2 [43] for CRISPR annotation. Genes that were predicted over an assembly gap and contained more than 50 % N were discarded. Protein annotation was performed with InterProScan v5.26–65.0 [44], priam vMarch 2015 [45] (together with legacy blast v2.2.26 [37]) and dbCAN v5.0 [46]. Additional EC (Enzyme Commission) numbers were derived via the GO (Gene Ontology) terms [47] derived from the InterProScan output. If an EC number could be assigned to a protein sequence, it was annotated with the canonical name of its EC number. Otherwise, all InterProScan domain names were searched for terms relating to functions involved in virus replication, sporulation, ribosomal proteins, CRISPR or any ‘subunit’ containing names, and these terms were used with priority for the naming. If this did not lead to any result, all domain names were searched for words ending in ‘ase’, indicating potential enzyme functions. Otherwise a random domain name was picked. All these annotation steps were carried out while disregarding generic or uninformative terms (e.g. containing ‘hypothetical’, ‘DUF’, ‘uncharacterized’). Domains containing these words were only considered after all other steps did not lead to any result. All programs were executed with standard parameters, unless specific parameters are mentioned.

### CRISPR analysis

CRISPR elements were predicted for all genomes with the CRISPR recognition tool v1.2 [43]. A blast database was built for all the different ECE groups based on the pyANI analysis. A blast search of all the CRISPR spacers against these databases was performed with the options -task blastn-short -outfmt ‘6 std qlen’, and afterwards all hits were filtered for having at least a match of 90 % of the query length.

### Data accessibility

All reference plasmid assemblies have been uploaded to the European Nucleotide Archive under accession numbers LR594541–LR594546 and LR595854.

## Results

### ECEs are abundant in *C. difficile*

There is substantial evidence that plasmids are more abundant in *C. difficile* than expected on the basis of the published number of characterized plasmids from this organism [16–19]. We set out to determine the prevalence and identity of ECEs in *C. difficile* in a high-throughput manner. To this end, we analysed public whole-genome sequence data from the NCBI. A total of 5403 samples Table S1See in the sequence read archive that were sequenced on Illumina machines in paired-end mode were processed using an in-house pipeline (Fig. 1) based on PLACNETw [26]. Of these, 5336 genomes were successfully assembled. In total, we identified 1066 putative ECEs within 692 genomes, which corresponds to a prevalence of 13 % (Table S2). These data confirm that ECEs are abundant in *C. difficile *.

**Fig. 2.**
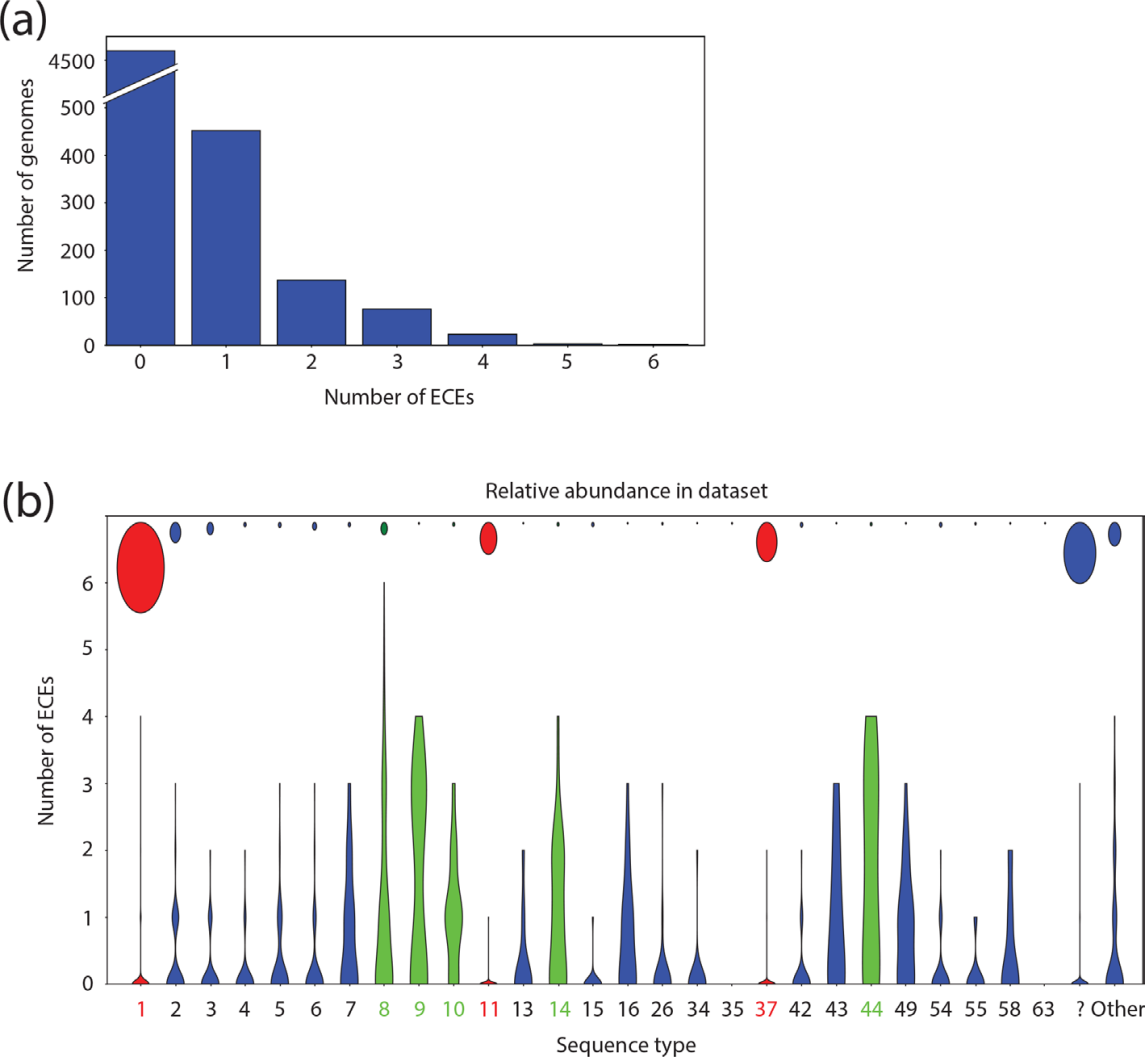
ECEs of *C. difficile *. (a) Number of ECEs per strain. (b) Distribution of the number of ECEs amongst various STs of *C. difficile *. The violin plots indicate the distribution of plasmids for a particular ST (in the figure ST1/1* is indicated as ‘1’), whereas the circles indicate the relative number of sequenced genomes for a particular ST. Only STs with 20 or more sequenced genomes are displayed separately. All other STs are summarized under ‘other’. Genomes to which no ST could be assigned are summarized under ‘?”. The STs belonging to the hypervirulent ribotypes RT27 (ST1) and RT078 (ST11), as well as the widely sequenced ST37, are marked in red. STs with more than one plasmid per genome on average are marked in green.

Most (451) of the genomes contained a single ECE, but the presence of two or three elements was also common (137 and 76 genomes, respectively) (Fig. 2a), with only 28 genomes carrying four or more ECEs. The highest number of ECEs observed for a single genome was six. This indicates that at least some of the ECEs are compatible with others.

### ECE content differs for different multilocus sequence types (STs)

Above, we observed variation in the number of ECEs per genome sequence. We wanted to establish whether certain types of *C. difficile* were more likely to contain ECEs than others. High-quality whole-genome sequence data allow the determination of STs. We could successfully assign a ST to 52 % (2770/5336) of the genome sequences using MLSTcheck (Table S1). A substantial number of genomes could not be assigned a ST (*n*=914), or were designated as closely related to an identifiable ST (*n*=1652) using this tool. For the analyses hereafter, we will refer to the latter group using their closest ST (e.g. ST1 indicates ST1 only, whereas ST1/1* indicates both ST1 and STs closely related to ST1).

Whole-genome sequencing data are biased towards clinical isolates as these are most frequently investigated. This is reflected in the relative abundance in our dataset of the well-known epidemic PCR ribotypes RT027 and RT078, which were the subject of extensive whole-genome sequencing studies [5, 9]. RT027 and RT078 belong to ST1 (clade 2) and ST11 (clade 5), respectively. A total of 1352 genomes were assigned to ST1/1* and 477 to ST11/11*. The next largest group (586 genomes) corresponds to ST37/37* (which includes the toxin A negative PCR RT017, clade 4). Together, these three groups make up 45 % of all the sequences analysed. Despite being so widely sequenced, ST1/1*, ST11/11* and ST37/37* contained only 62, 4 and 6 ECEs, respectively, of the 1066 elements identified here (Fig. 2b, Table S3).

Further analysis suggests that most of the ECEs in ST1/1*, ST11/11* and ST37/37* are in fact likely bacteriophages. These are in part derived from technical spike-in controls (phiX174) [48]: 21/62 of the ST1/1*, 0/4 of the ST11/11* and 1/6 of the ST37/37* ECEs correspond to this phage. *Clostridium* phage phiCD38-2 [49] was also common: 20/62 ST1/1*, 1/4 ST11/11* and 4/6 ST37/37* ECEs correspond to this phage. Finally, in ST11, *Clostridium* phage phiCD6356 [50] was identified once. Overall, 48/72 ECEs in these epidemic types are likely to be phage, further reducing the number of putative plasmids in these groups. Notably, the majority of ST1/11/37/* isolates do not contain any ECEs, suggesting a possible negative correlation between ECE carriage and epidemicity.

By contrast, we noticed that certain STs more frequently contain ECEs. For example, ST8/8* contained 189 ECEs in 183 analysed genomes, with 1–6 per genome (Fig. 2b, Table S3). ST8 includes RT002, the seventh most common PCR ribotype in Europe [51]. Other STs that appear to contain at least one ECE on average are ST9/9*, ST10/10*, ST14/14* and ST44/44* (Fig. 2b, Table S3). Notably, all these STs fall in clade 1 [52, 53]. The highest mean ECE content was observed for ST9 with 39 elements in 15 samples (2.6 per genome), followed by ST44 (2.2 per genome).

Taken together, our data suggest that certain *C. difficile* types may be more tolerant to plasmid carriage than others. pCD630- and pCD6-like plasmids are common.

We wanted to confirm that our pipeline can identify *bona fide* plasmids. Therefore, we screened the identified ECEs against known *C. difficile* plasmid sequences, like pCD6, pCD630 and others (see Methods). We found several plasmids highly similar and sometimes identical to these known ones, derived from various STs. The class pCD-WTSI1/pCD630-like plasmids contained 378 plasmids (the majority more similar to pCD-WTSI1), and 189 plasmids were similar to pCD6 (these were contained in a larger family of 296 ECEs, see ECE6 below). We also identified various ECEs overlapping with the previously identified phages and megaplasmids (*n*=70) [17].

Interestingly, we inadvertently also identified a replicative cloning plasmid carrying a chloramphenicol/thiamphenicol-resistance gene in one of the whole-genome sequences (ERR125924). Plasmids are generally introduced as shuttle vectors from the hosts *Escherichia coli* [14] or *Bacillus subtilis* [54]. We screened all ECEs for regions required for transfer from the conjugation donor (*traJ*/*oriT* or the Tn*916 oriT*) and replication in Gram-negative bacteria (pBR322/ColE1 origins), but did not find any further cloning vector contamination.

Overall, our data confirmed that our analysis does in fact detect *bona fide* plasmids, as well as certain phages. Plasmids with significant homology to the characterized plasmids pCD630 [15, 16] and pCD6 [14] were the most common (Table S2).

### Identification of six novel families of ECEs

**Fig. 3.**
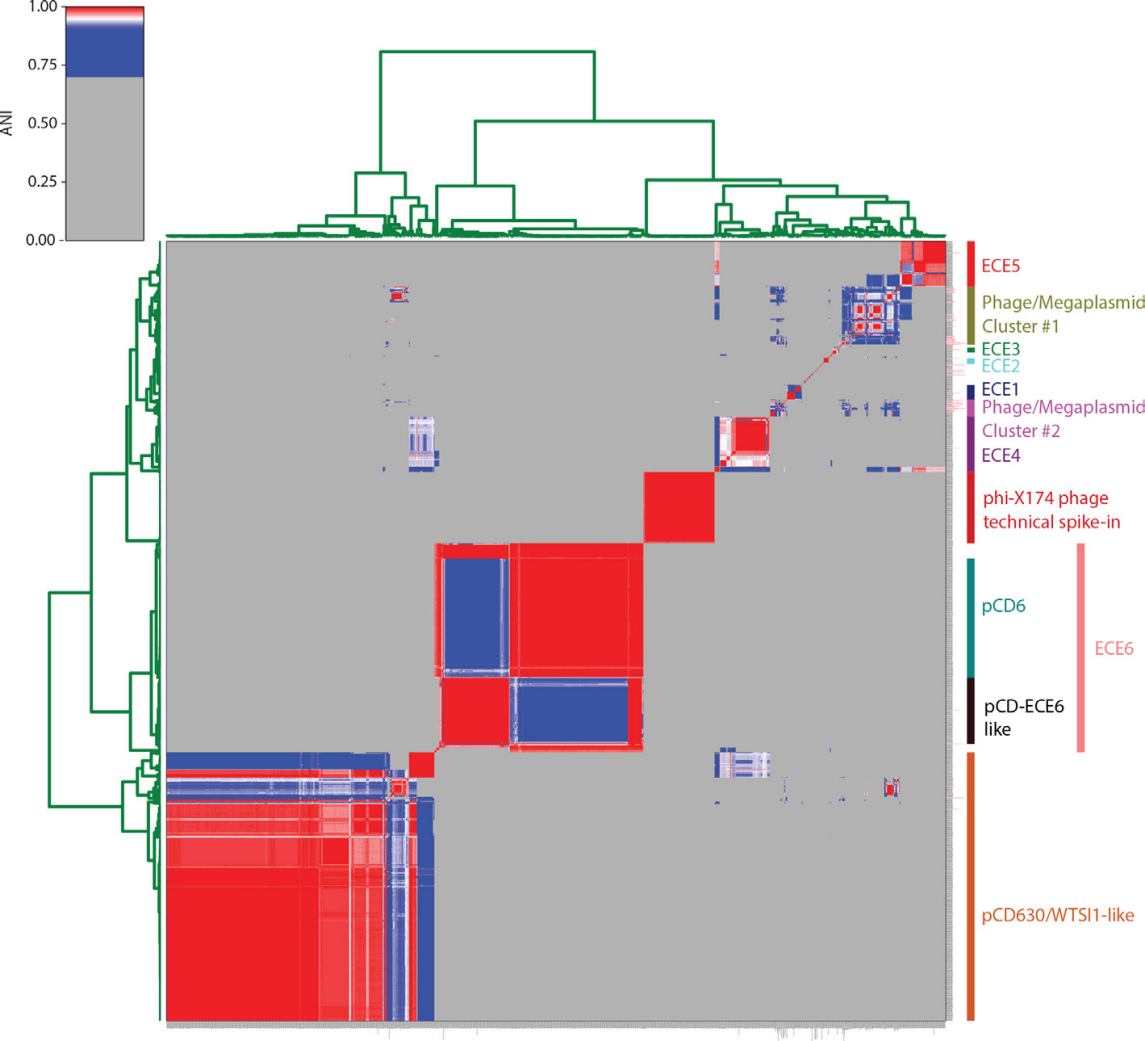
Identification of ECE families. Hierarchical clustering based on ANI was performed. Red to blue colours indicate similarity. Grey values indicate that no significant similarity was found. The names of the clusters are given on the right. A high-resolution version of this figure (with readable plasmid names) is provided as Fig S1.

Many of the ECEs identified do not have homology to the previously described phages and plasmids discussed above (*n*=222), or share only limited homology (*n*=107). We reasoned that those ECEs that are part of a homologous group are more likely to represent legitimate *C. difficile* plasmids and, therefore, clustered the ECEs by sequence similarity (Fig. 3) . This resulted in the identification of six putative homology groups, further termed ‘families’, of ECEs (*n*=478), and a group of singletons (*n*=40) (Table S2). Some details of the different families are discussed below.

#### ECE1

**Fig. 4.**
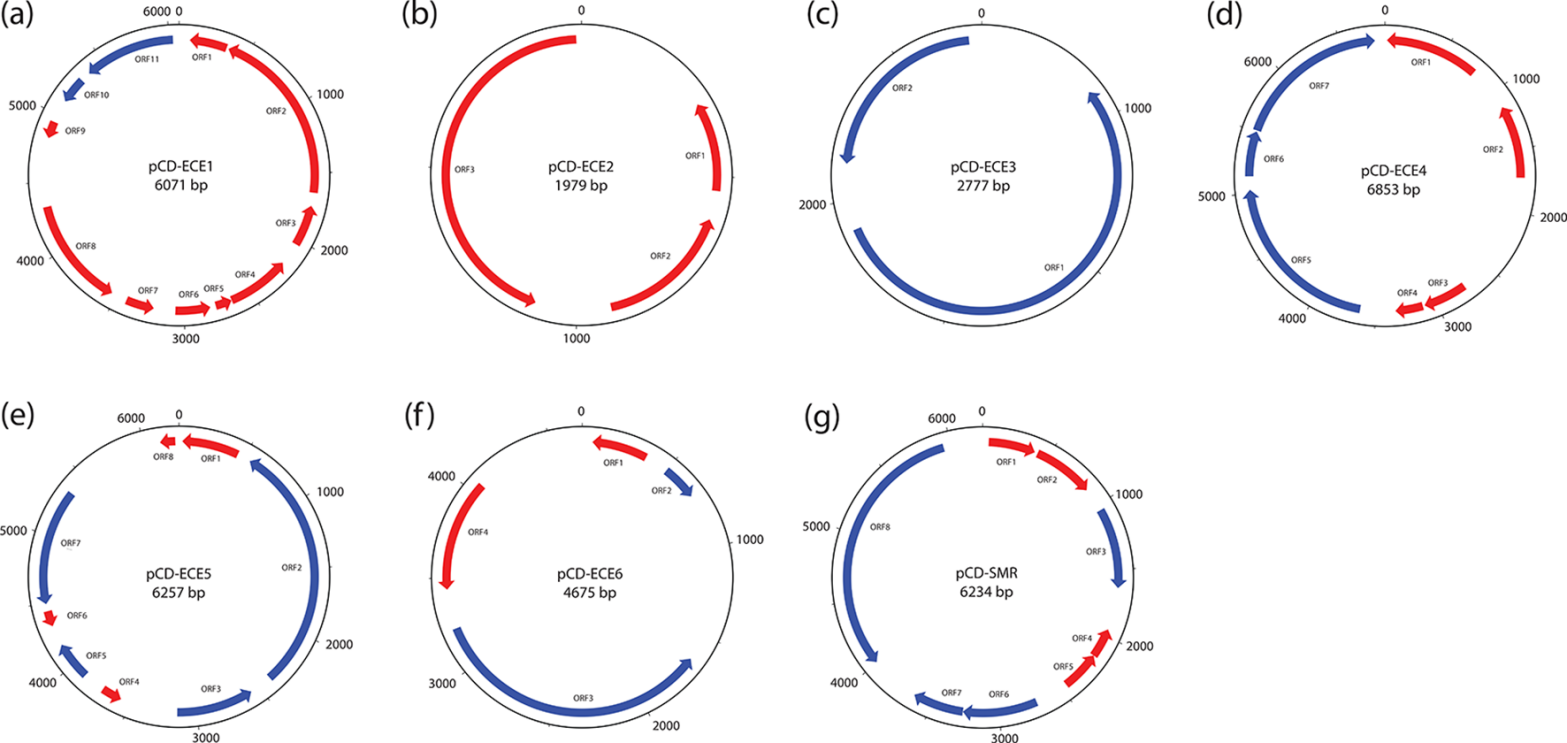
Maps of ECEs. Schematic diagram of representative members of the six putative plasmid families, pCD-ECE1 (a), pCD-ECE2 (b), pCD-ECE3 (c), pCD-ECE4 (d), pCD-ECE5 (e) and pCD-ECE6 (f), and the singleton putative multidrug-resistance plasmid pCD-SMR (g). Tick marks indicate 500 bp intervals, with every 1000 bp labelled. Red ORFs are hypothetical (no results in the automated annotation pipeline, see Methods). Blue ORFs indicate genes for which a putative function could be assigned in the automated annotation, as follows. (a) ORF10, Arc-type ribbon-helix-helix protein; ORF11, zonular occludens toxin. (b) No functions annotated. (c) ORF1, plasmid replication protein; ORF2, plasmid recombination enzyme. (d) ORF5, plasmid recombination enzyme; ORF6, penicillinase repressor; ORF7, BlaR1 peptidase M56. (e) ORF2, type III restriction enzyme (res subunit); ORF3, phage integrase family protein; ORF5, HNH endonuclease 5; ORF7, DNA-directed DNA polymerase. (f) ORF2, lambda repressor-like, DNA-binding domain superfamily protein; ORF3, alpha-helical domain, primase C-terminal like. (g) ORF3, winged-helix-like DNA-binding domain superfamily protein; ORF6, bacterial regulatory protein, TetR family; ORF7, small multidrug-resistance protein; ORF8, MobA/MobL protein.

The 6.1 kb type plasmid pCD-ECE1 (LR594544.1) is derived from ERR1015479 (Fig. 4a). ECEs in this family ranged in size between 6071 and 7284 bp and appeared in 21 samples. The distribution of STs showed some clustering, with seven samples belonging to ST436, and coming from the BioProject PRJEB5486, where eight samples were sequenced. The eighth sample of this BioProject also contained this plasmid, but it was assigned to ST9 (and potentially contaminated with a *Lactobacillus *). Another 10 samples belonged to ST9, 2 to ST10 and 1 to ST75*. A total of 9 of these 21 sequences were predicted to be circular, with eight of these having a length of 6071 bp and a minimum identity over the full length of 99.9 %.

#### ECE2

The 2.0 kb type plasmid pCD-ECE2 (LR594542.1) is derived from ERR251819 (Fig. 4b). ECEs in this family appeared in seven samples belonging to five STs from five different BioProjects. Six of these were assembled into a single contig of 1979 bp, and were due to overlaps confirmed to be circular. Nearly all ECEs showed 100 % identity, with only a single SNP in one of the ECEs. The seventh ECE was fragmented into multiple contigs, which overlapped with each other, with a cumulative non-redundant sum of 1979 bp. Also, this ECE showed a minimum 99 % identity to the other sequences. Functional annotation only showed three hypothetical proteins on each ECE. No significant homology to any sequence in the NCBI database could be found.

#### ECE3

The 2.8 kb type plasmid pCD-ECE3 (LR594543.1) is derived from ERR125924 (Fig. 4c). ECEs in this family were found in ten samples, of which five belonged to ST1/1*. Four of these were predicted to be circular, and their size varied between 2.8 and 6.8 kb. Two ECEs, derived from different STs within the same BioProject, were circular and 100 % identical with a size of 3361 bp. They showed a total homology of >2000 bp with an identity of 94–95 % to the reference sequence. Homology was less strong for the fourth circular ECE, which had an identity of only 86–88 % over a length of <2000 bp. Functional annotation only identified plasmid replication and recombination proteins, in addition to hypothetical proteins, and in one case a DNA polymerase.

#### ECE4

The 6.9 kb type plasmid pCD-ECE4 (LR594545.1) is derived from ERR347487 (Fig. 4d). The 79 ECEs in this family were found in samples from more than 10 different STs from various BioProjects. The size ranged from 5 to 22 kb. These ECEs included 4 identical circular sequences with a size of 15 kb, belonging to ST9/9* from 2 different BioProjects, and 21 circular sequences with a size of 6853 bp, belonging to >5 BioProjects and STs, and a few other identical sequences with varying sizes. The diversity of sizes in the ECE4 group, based on our pyANI analysis, is striking. It is possible that more detailed characterization will reveal the existence of subfamilies or multiple families that have been grouped based on our method.

#### ECE5

The 6.3 kb type plasmid pCD-ECE5 (LR594541.1) is derived from ERR340291 (Fig. 4e). This family contained 65 ECEs from various STs and BioProjects. The size ranged from 5044 bp up to 12 kb. This class included 15 predicted circular sequences with a size of 6257 bp (present only in STs 6, 7 and 8/8*), 8 circular sequences of 6651 bp (belonging to ST 9, 12 and 436) and 4 circular sequences of 5819 bp.

#### ECE6

The 4.7 kb type plasmid pCD-ECE6 (LR594546.1) is derived from ERR251831 (Fig. 4f). This family included the well-characterized pCD6 and comprised 296 plasmids. We did not group plasmids with high similarity to pCD6 separately, as they could not be clearly distinguished from the other plasmids in the pyANI analysis (Fig. 3). It could be divided into three subfamilies: a small group (including ERR340281.plasmid_1, *n*=20) seemingly consisting of parts of both other subgroups, a group that includes pCD6 and a further not yet characterized type of plasmid (pCD-ECE6). On the basis of the pyANI analysis, these subfamilies clearly grouped together (Fig. 3). The size ranged from a single plasmid of 3335 bp (16 contigs), through a group of circular plasmids with a size of 4675 bp [*n*=45, belonging to various (>5) BioProjects and STs], up to a size of 18 kb. Most of these sequences could not be confirmed to be circular, but various instances with varying sizes appeared in more than one sample with >95 % identity. The largest that could be circularized had a size of 8297 bp.

#### 

These six classes represented 478 of the 1066 identified ECEs. As indicated above already, the known class of pCD-WTSI1/pCD6-like plasmids contained 378 plasmids (the majority more similar to pCD-WTSI1). The technical spike-in controls (phiX174) contained another 100 sequences, validating that our approach detects elements that are not linked to the chromosome. In total, 70 sequences were assigned to probable *Clostridium* phages and megaplasmids (pCDBI1, pAK1, pAK2, etc).

A total of 40 ECEs did not fall into one of these families (singletons); these may represent legitimate low-prevalence ECEs in our sample population or may reflect incidental contaminations. Two known plasmids could not be classified into one of the groups either, pZJCDC-S82 (JYNK01000020.1) and pCd13_cfrC (MH229772.1), indicating that low-prevalence plasmids do exist in the population.

#### Function of new ECE families

We annotated all these newly identified ECEs to be able to analyse their function. Many of the genes were annotated as encoding hypothetical proteins. Of the genes that had a function assigned, mostly functions related to DNA processing and plasmid replication (e.g. RNA polymerase, winged helix-like DNA-binding domain superfamily, helicase, resolvase, zinc fingers) or phage elements (e.g. integrase, phage tail, phage capsid) were present. Some of these functions and their relationships to different plasmid classes are detailed below.

##### Identification of putative plasmid maintenance functions

The mode of replication for the newly identified ECEs is unknown. In an attempt to identify the origin of replication, the sequences of pCD-ECE1 to pCD-ECE6 were analysed using DoriC [55] and PlasMapper [56], but this did not yield any putative origin sequences. As origins and replication initiation proteins are frequently adjacent to one another, we performed structural modelling of ECE ORFs using Phyre2 [57]. This identified multiple proteins with a likely role in plasmid maintenance. In pCD-ECE1, the amino-terminal domain of the ORF8-encoded protein shows homology to the DNA binding domain of REP, a viral DNA replication initiation protein (PDB 1L5I; confidence 71.7, 20 % identity). In pCD-ECE2, the protein encoded by ORF3 is highly similar to the rolling circle replication initiator proteins from *Staphylococcus aureus* (PDB 4CWC; confidence 100, 19 % identity) and *Geobacillus stearothermophilus* (PDB 4CIJ; confidence 99.6, 20 % identity). The replication of pCD-ECE3 is likely dependent on ORF2, encoding a protein with the strongest structural homology to the RepB replication initiator protein of plasmid pMV158 (PDB 3DKX; confidence 100, 31 % identity). Notably, this protein is predicted to contain a REP domain similar to that of pCD-ECE1 ORF1. For pCD-ECE5, Phyre2 predicts that the protein encoded by ORF7 is the initiator, based on homology with pi initiator protein of plasmid R6K (PDB 2NRA; confidence 100, 23 % identity) and RepE of F plasmids (PDB 2Z9O; confidence 100, 21 % identity). Finally, for pCD-ECE6, ORF3 appears to encode a PRIMPOL protein that could be responsible for replication based on clear structural homology with RepB’ of RSF1010 (PDB 3H25 and 3H20; confidence >99, 15–17 % identity). We could not predict likely replication initiators in pCD-ECE4 and pCD-SMR (see below). We also noted that the structure predictions for multiple other proteins suggest DNA binding activity, and some of these might be relevant for plasmid maintenance. For instance, ribbon-helix-helix proteins, such as those encoded by pCD-ECE1 ORF7 and ORF10, can be either toxin–antitoxin systems involved in plasmid stability or CopG-like proteins that regulate copy number. The exact function of these proteins awaits experimental validation.

##### Novel ECE families may encode virulence factors

pCD-ECE1 belongs to a family of ECEs that is characterized by the presence of an Arc-type ribbon-helix-helix domain (IPR013321; ORF10) and an ORF with homology (30 % identity, 41 % similarity over 29 % of the *C. difficile* protein length) to the UniProt protein P38442 encoding the zonula occludens toxin (Zot) (IPR008900; ORF11) (Fig. 4a). This annotation is further supported by a Phyre2 structure prediction [57] that identifies structural homology with the amino-terminal domain of zonula occludens toxin from *Neisseria meningitidis* (PDB 2R2A; 100 % confidence, 17 % identity) over nearly the full length of the *C. difficile* protein. Zot is an enterotoxin from bacteriophages infecting Proteobacteria, including enteropathogens such as *Vibrio* and *Campylobacter* spp., that increases intestinal permeability by affecting tight junctions, and a relation with inflammatory bowel disease has been suggested [58, 59]. Indeed, one of the ECEs from this family contained a gene encoding a phage capsid protein (IPR008020), which was not seen for the other sequences. Notably, this sequence showed only partial homology to the other ECE1 sequences, with identities ranging between 84–93 % over 2182 bp. Other obvious phage elements could not be identified in these ECEs. The contribution of this putative toxin to *C. difficile* infection, when carried on a plasmid, is currently unknown.

##### Novel ECEs may encode antibiotic-resistance determinants

A singleton ECE (not falling into one of the ECE families described above) was identified as carrying a putative antibiotic-resistance determinant. This circular 6.2 kb plasmid, dubbed pCD-SMR (derived from ERR125942; accession ERZ940801, ST1; Fig. 4g) contains a gene encoding a TetR-family regulator (IPR001647; ORF6) adjacent to a small multidrug-resistance protein (IPR000390; ORF7). Both of these functions are rather generic and also often appear at genomic locations that are not related to drug resistance. In this particular case, however, the transporter shows homology to the multidrug efflux transporter EbrB from *B. subtilis* (UniProt P0CW82; 46 % identity over 89 % of the *C. difficile* protein length) and to the multidrug efflux transporter EmrE of *E. coli* (UniProt P23895; 40 % identity over 90 % of the *C. difficile* protein length). It also contains a MobA/MobL mobilization protein (IPR005053; ORF8), and a winged helix-like DNA-binding domain superfamily protein (IPR036388; ORF3). A region of ~1 kb, containing part of the regulator and the drug transporter, may be derived from a *Listeria* transposon or plasmid pK5 from an uncultured organism (KJ792090.1).

Another singleton ECE (derived from sample ERR247053, ST48; Table S2) was identified as containing a protein with the motif for an aminoglycoside-2''-adenylyltransferase (IPR019646). It shows the highest homology to a lincosamide-resistance protein from *Staphylococcus haemolyticus* (UniProt P06107; 55 % identity over 99 % of the *C. difficile* protein length). The ECE was assembled in nine contigs with a cumulative size of 5384 bp, only containing one more annotated ORF, encoding a mobilization protein. The contigs showed a high identity (>93 %) to various genomic and plasmid regions of *Campylobacter jejuni *.

The majority the plasmids belonging to the ECE4 and ECE6 families (pCD6-like) carry the genes BlaR1 (IPR008756) and BlaI (IPR005650) (62/79 ECE4 both genes, 197/296 BlaR1 in ECE6 and 194/296 BlaI in ECE6). While these do not confer resistance to any antibiotics, they regulate the induction of β-lactamases in various species [60, 61], including *C. difficile* [62]. The genes found on ECE4 and ECE6, however, show only limited similarities (20–30 %) to the chromosomal regulators identified in a another study [62]. Therefore, the significance of the carriage of these genes for resistance to β-lactams is currently unknown.

##### An identified ECE potentially encodes a bacteriocin

One singleton ECE (derived from sample ERR125936, ST unknown; Table S2) contained many functional domains unique to our ECE dataset. This ECE contained three contigs with a total size of 18 812 bp, and 20 ORFs. Despite very limited homology at the nucleotide level, most protein sequences showed homology to protein sequences derived from a single contig of approximately 19 kb from an unknown *Muribaculaceae* species (RIAY01000031.1). The domains of interest include a peptidase of the C39 family, a TonB-dependent receptor, a ubiquitin-activating enzyme, an acyl-CoA *N*-acyltransferase, a protein with a generic prokaryotic membrane lipoprotein attachment site and a radical SAM enzyme (potentially split into two coding sequences over two contigs). We suggest this cluster to be involved in bacteriocin synthesis for three reasons. First, the InterPro descriptions of the peptidase (IPR005074) and the radical SAM enzyme (IPR023885) indicate a possible involvement in bacteriocin processing and biosynthesis of ribosomally synthesized and post-translationally modified peptides (RiPP) [63], respectively. Second, an analysis with Bagel4 indicated that the ubiquitin-activating enzyme had homology to a putative bacteriocin biosynthesis protein from *Streptococcus thermophilus* (Q5LXQ2), and labelled the contig as potentially containing a sactipeptide [64]. Finally, TonB-dependent receptors, lipoproteins and radical SAM enzymes are common components of bacteriocin biosynthesis clusters and acylation is often observed during bacteriocin biosynthesis [65, 66]. While no specific bacteriocin gene could be pinpointed, the presence of most of the unusual genes on this ECE can be explained the presence of such a RiPP gene cluster.

##### CRISPR spacers mainly target phage, not novel ECE families

Since some of the ECEs were identified as phages, we wondered whether CRISPR-based resistance mechanisms against ECEs exist, and whether these do or do not correlate with ST. We predicted 371 008 CRISPR spacers in all genomes (*n*=3994; in 1340 genomes no CRISPR spacers could be predicted), making a total of 31 968 unique CRISPR spacers. The highly sequenced STs ST1/1*, ST11/11* and ST37/37*, which do not seem to carry many ECEs (Fig. 2b), showed on average a medium (94), high (134) and low (35) carriage of CRISPR spacers. The MLST types that seem on average to carry ECEs (ST8/8*, ST9/9*, ST10/10*, ST14/14*, ST44/44*), showed medium and high numbers of CRISPRS (84, 83, 105, 127 and 127, respectively). Thus, we did not see a clear relationship between ECE carriage and the number of CRISPR spacers.

Furthermore, we tried to match the CRISPR spacers against the various ECEs. We found only 5 CRISPR spacers (of 31 968 unique CRISPR spacers) that matched against the technical spike-in phiX174 phage, indicating that most of the prediction did not cause false-positive artefacts. In 110 cases, CRISPR spacers matched against ECEs isolated from their own genome. A total of 57 of these 110 were directed against ECEs of the class phiCD38-2, 32 against the pCD630/pCD-WTSI1 family and 10 against ECE1. Thus, only a minor fraction of CRISPR spacers targets endogenous ECEs.

Most CRISPR spacers appear to target the two classes of phage/megaplasmids (7387 and 3496 CRISPR spacers, respectively), whereas many fewer targeted the six novel ECE families (ECE1–ECE6) (934, 29, 0, 112, 13, 148, respectively) or the pCD630/pCD-WTSI1 family (*n*=978). Moreover, the phage/megaplasmid-targeting CRISPR spacers seemed to be more broadly distributed (the most widespread CRISPR spacers for both clusters predicted in 651 and 711 genomes) than the CRISRP spacers targeting the other ECE classes (most widespread: 575, 64, 498, 22, 524 and 632 for ECE1, 2, 4, 5, 6 and pCD630/pCD-WTSI1, respectively). Together these data suggest that CRISPR-mediated defence likely does not play a major role in resistance against the ECE families identified here.

## Discussion

Here, we present the first comprehensive *in silico* survey of ECEs in *C. difficile *. Our major findings are that ECEs are abundant (present in ~13 % of all genomes analysed), strains can simultaneously carry 2–6 ECEs from different families and that there appear to be at least six families of ECEs that have not been previously characterized. The classification into families should direct and facilitate future functional analysis of the ECEs. For instance, epidemiological analyses and cloning of the putative toxin in a *C. difficile* shuttle vector and comparison of isogenic toxin-carrying and non-toxin carrying strains will elucidate whether Zot can contribute to disease severity in humans and animal models.

Our study has several limitations. First, due to the use of publicly available sequencing data without access to the strains from which these data were generated, we are unable to confirm that the identified ECEs are in fact plasmids. However, our work allows the identification of conserved regions in each ECE family, which can be further developed into ECE-specific PCRs to screen available collections of strains. The ECE content of PCR-positive strains can then be verified using established methods [16, 17]. Next, due to a bias in the available genome sequences towards clinical isolates of specific PCR ribotypes, our analysis may fail to capture the full breadth of plasmids that are present in *C. difficile *. However, one might argue that for clinical relevance, the current collection should provide ample information. More targeted sequencing strategies, aimed at a greater diversity of *C. difficile* strains from underrepresented sources and geographical locations, may lead to the identification of more ECEs. Furthermore, the produced assemblies did not undergo any quality control, and are partially of substandard quality. The low quality assemblies fall mostly in the category to which no ST could be assigned and, therefore, does not affect the analysis of genomes with a ST assigned. Since our identified plasmids are mainly of small size (<20 kb), and the majority belong to one of the previously or newly identified classes, we do not expect that many of these are false positives. We rather expect that due to the poor quality, some of the low-copy megaplasmids might have been incorrectly lumped into the genome, and that the actual diversity in ECEs might be even greater than what we discovered. Finally, we note that the *in silico* methodology employed here clearly also identifies phages, which can exist as extrachromosomal dsDNA [20, 21, 67] (see for instance the phage/megaplasmid cluster in Fig. 3). Nevertheless, plasmids may encode phage proteins (incomplete phages) as the result of recombination between plasmids and phage [16, 17, 68]. Though we show that our approach can accurately identify *bona fide* plasmids, establishing whether the identified ECEs are phage, plasmid or an intermediate may need additional experimentation.

An intriguing finding is that *C. difficile* can harbour multiple ECEs simultaneously in the absence of selection. Indeed, the finding that 630Δ*erm* can harbour both pCD6-based replicons and the native pCD630 plasmid [16] supports this notion. Our work identified a ST8 strain that contained six ECEs; these included a pCD6-like plasmid, a pCD-WTSI1-like plasmid (from the pCD630 family) and pCD-BI1-like plasmid. This suggests that there is no plasmid incompatibility, or that the co-occurring plasmids do not belong to the same compatibility group. So far, no experiments have been described that make use of the simultaneous introduction of two plasmids with different replicons into *C. difficile *. Moreover, to our knowledge, genetic manipulation of *C. difficile* has been limited to a small set of PCR ribotypes to date (RT012, RT027 and RT078), and the efficiency of the different replicons has been found to differ between these ribotypes [69]. Therefore, the ECE pool described here represents an interesting possibility to adapt or expand existing tools for use in a broader range of *C. difficile* types.

For conjugative elements, transfer between *C. difficile* is well established [22] and transfer of the pathogenicity locus via a so far uncharacterized mechanism has also been described [70]. For the ECEs identified in this study, it is unknown whether they are transferable between strains. Though conjugative plasmids are generally larger than mobilizable plasmids [71], large plasmids in *C. difficile* are likely to be non-conjugative due to the absence of conjugation or mobilization functions [17]. In general, the ECEs identified in the present study are smaller (967 of 1066 ECEs are <20 kb) and certain ECEs do seem to encode proteins relevant for mobilization or conjugation (such as ORF8 of pCD-SMR, Fig. 4g). It remains to be established whether these functions allow interspecies or intraspecies transfer from *C. difficile *﻿, and if so, what other requirements for transfer may exist.

Overall, this work provides a starting point for investigations into the role of plasmids in *C. difficile* physiology. Additionally, it opens up the possibility of generating novel cloning vectors that may be particularly suitable for the manipulation of one or more *C. difficile* types.

## Data bibliography

Accession numbers for all publicly available sequence data used in these analyses are described in Table S1. PDB: 1L5I, 4CWC, 4CIJ, 3DKX, 2NRA, 2Z9O, 3H25, 3H20, 2R2A. UniProt: P38442, P0CW82, P23895, P06107.

## Supplementary Data

Supplementary File 1

Supplementary File 2
