# Supplementary material for: An in silico survey of Clostridioides difficile extrachromosomal elements
Source: Microb Genom. 2019 Sep 17;5(9):e000296. doi: 10.1099/mgen.0.000296 (PMC6807378; doi:10.1099/mgen.0.000296)
Supplement: Supplementary File 2 [file mgen-5-296-s002.pdf]

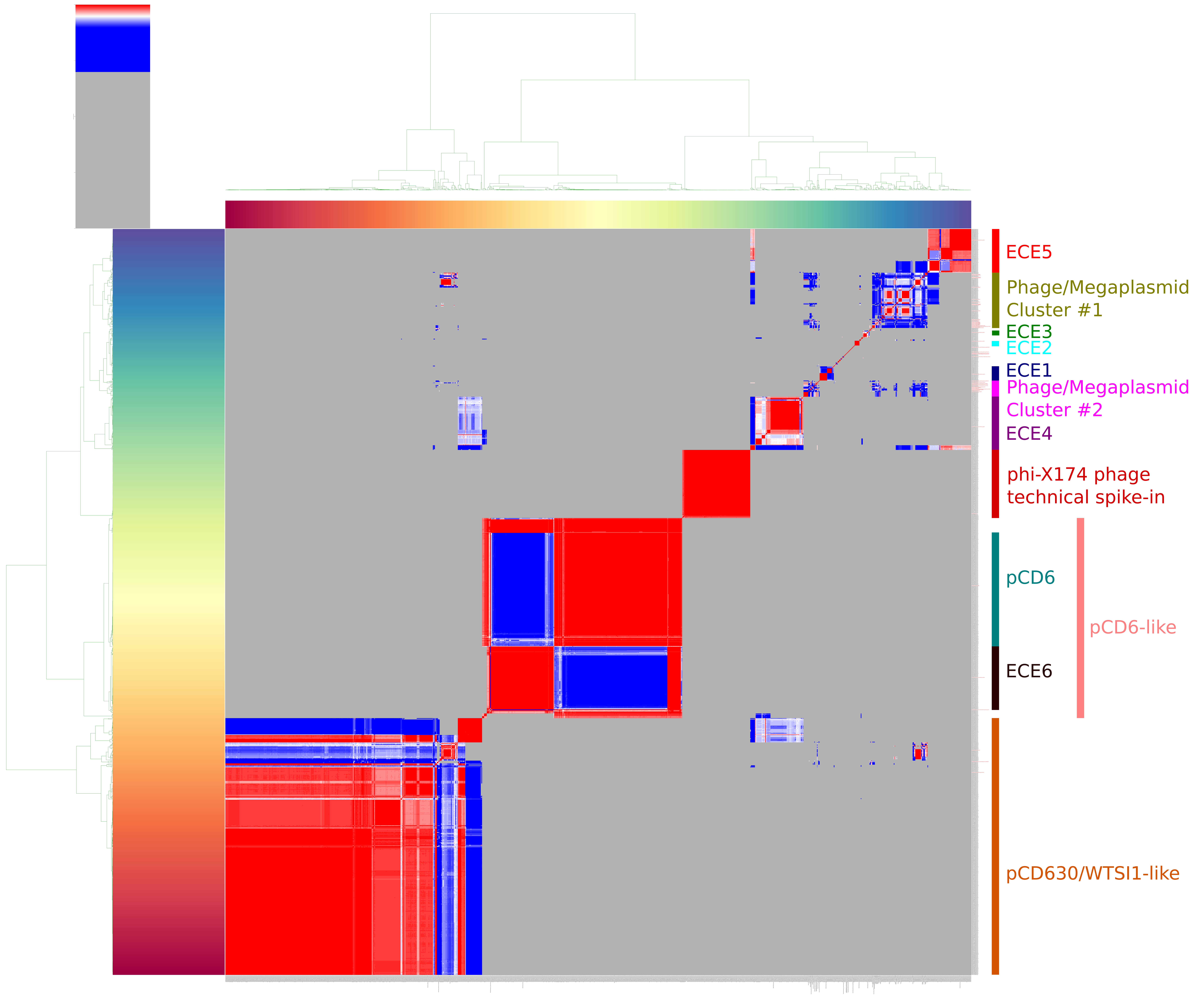
ECE5
Phage/Megaplasmid Cluster #1
ECE3
ECE2
ECE1
Phage/Megaplasmid Cluster #2
ECE4
phi-X174 phage technical spike-in
pCD6
pCD6-like
ECE6
pCD630/WTSI1-like
